# Tests of Physicochemical and Mechanical Strength Properties of Polymer Composites on an Epoxy Resin Matrix, Modified by a Constant Magnetic Field

**DOI:** 10.3390/ma15196730

**Published:** 2022-09-28

**Authors:** Ewa Miękoś, Michał Cichomski, Marek Zieliński, Tomasz Klepka, Dariusz Sroczyński, Anna Fenyk

**Affiliations:** 1 Department of Inorganic and Analytical Chemistry, Faculty of Chemistry, University of Lodz, Tamka 12, 91-403 Lodz, Poland; 2 Department of Materials Technology and Chemistry, Faculty of Chemistry, University of Lodz, Pomorska 163, 90-236 Lodz, Poland; 3 Department of Technology and Polymer Processing, Faculty of Mechanical Engineering, Lublin University of Technology, Nadbystrzycka Street 36, 20-618 Lublin, Poland

**Keywords:** polymers, composites, constant magnetic field (CMF)

## Abstract

The aim of the research presented in the paper was to obtain new polymer composites with strong functional properties on the Epidian 5 epoxy resin matrix. The polymer composites contained admixtures of expanded graphite, powder graphite, birch bark containing botulin, and yellow dextrin in set amounts of 20% by weight. Their various mechanical parameters and physicochemical properties were investigated. The research involved determining the effect of a constant magnetic field with magnetic induction B, under the influence of which the parameters and properties of polymer composites have been changed. For example, in a constant magnetic field with an induction of B = 0.5 T there was an increase in the hardness of the composite with an admixture of birch bark from 24.01 to 26.96 N/mm^2^ (12.3%), or in the composite with the addition of yellow dextrin from 26.12 to 29.93 N/mm^2^ (14.6%). It was also found, for example, that the water absorption of the resin itself decreased from 0.18% to 0.13%, and the composite with graphite powder from 0.48% to 0.46%. Changes in these parameters, often beneficial, may be important in terms of potential application of those new materials in industry as alternatives.

## 1. Introduction

Polymer composites are widely used, among other uses, in the chemical, construction, electrical, and household industries, and therefore a continuous increase in demand for this type of material has been observed for many years. On the other hand, production of new types of plastics leads to a significant increase in the amount of waste generated. Unfortunately, most of the known polymer composites are characterized by low biodegradability. Therefore, it has become extremely important to search for new materials with properties similar to those produced at present, but with better ecological parameters [1]. New polymer composites should also have certain properties, e.g., high mechanical strength, and certain specific features, e.g., the ability to conduct electricity and heat, as well as antimicrobial and magnetic properties. In the case of polymer magnetic composites, the decisive factor for their properties are interactions at the phase boundary of the magnetic particles/polymer matrix. Charles et al. developed a method for functionalizing carbonyl iron particles using epoxy silane [2]. The results of mechanical and electromagnetic tests showed that functionalization increased the rigidity, fracture resistance, and induction heating rate of the composites compared to composites containing non-functionalized carbonyl iron particles. Kobyliukh et al. have demonstrated that the introduction into the polymer matrix of nanoparticles of a hybrid filler based on graphene or carbon nanotubes with various forms of iron oxides improves both macro- and micro-properties of composites as a result of the synergistic action of the individual components [3]. The formation of an oriented filler network in the polymer matrix was also observed. Hosseini et al. used magnesium oxide (MgO) nanoparticles in 0–5% *w*/*w* amounts to improve the physicochemical properties of biopolymers [4]. The results of the research showed that after the introduction of magnesium oxide nanoparticles, there was an increase in the thickness of the films produced and a decrease in moisture content, water solubility, and water vapor permeability. In addition, biocomposite films showed greater antioxidant activity. Chen et al. in their study took advantage of the ability of carbonyl iron particles to absorb microwaves [5]. Owing to the developed method, carbonyl iron particles in the shape of flakes with increased microwave absorption were obtained. The use of dextrins as a polymer composite filler also resulted in an increase in the microwave absorption frequency range compared to polymer composite with magnetite nanoparticles as a filler. In addition, the increase in temperature caused an increase in microwave absorption [6]. Polymer composites with an admixture of graphene have antimicrobial properties with a wide spectrum of action, which is particularly valuable in combating the resistance of microorganisms to antibiotics and other drugs, dangerous for humans [7,8,9,10,11,12,13].

Another parameter affecting the properties of polymer composites is the use of an external constant magnetic field. The magnetic field has been found to increase the polymerization rate of fibrin fibers by 55–70% due to their orientation along the force lines of the magnetic field [14,15].

Kukhta et al. obtained composite films from graphene nanoplates and magnetite Fe_3_O_4_ both in the presence and without the presence of magnetic field. Poly(3,4-ethylenedioxythiophene):polystyrene sulfone acid was used as the stabilizing polymer matrix. The structure of composites obtained in a magnetic field was found to be more porous than that of composites obtained without the presence of a magnetic field. Additionally, thin films obtained without the use of a magnetic field demonstrated activation character of conductivity, whereas the conductivity of thin films obtained in the magnetic field was metallic in character. In other words, the magnetic field acting during the film’s formation caused the properties to change from semiconductor to metallic [16].

The presence of an external constant magnetic field can also be taken into account in the design of smart materials, thus obtaining materials with complex magnetically induced configurations and shapes [17].

Zhuang et al. investigated electrical and mechanical properties of cement-based composites with magneto-aligned nickel powder [18,19]. The research has demonstrated that in the case of composites obtained in the presence of a magnetic field, there was a significant decrease in electrical resistivity, and the percolation threshold was achieved at lower filler concentrations. The appropriate selection of magnetic induction B and the duration of the magnetic field has been also found to lead to an increase in the piezoresistivity of cement-based composites. Moreover, the proposed method made it possible to solve the problem of high filler consumption and low fluidity and strength of composites with randomly dispersed nickel particles.

An important direction of the research on biocomposites is to reduce the costs of their production. For this purpose, various waste materials, e.g., flax fibers, are used as fillers [20]. 

By replacing glass fibers with flax fibers and using a biodegradable thermoplastic matrix, biocomposites can be recycled and composted. Biodegradable flax-fiber-reinforced thermoplastics such as (poly-(hydroxyalkanoate), poly-(butylene-succinate) and poly-(lactide) were investigated and their properties compared with poly-(propylene) and maleic-anhydride grafted poly-(propylene). It was found that the mechanical properties of biodegradable polymer/flax composites were comparable to, or better than, those of polypropylene/maleic anhydride/flax composites, and the main factor determining the mechanical properties was adhesion at the fiber-polymer matrix interface [21].

Introducing such hard-to-process waste into the polymer matrix allows the creator to obtain original material compositions and products with original properties. They can be used, for example, as covers of electrical components in order to shield from electromagnetic radiation, energy dissipation, or heat reception, in addition to maintaining heat at the required level, depending on the amount of filler. These products are easily biodegradable, and at the same time, they retain good mechanical properties. Scientific research in the field of polymers is the driving force behind the progress of civilization. Additionally, current research on the synthesis of new polymeric materials increasingly provides the sophisticated solutions for virtually all fields of technology and economic development. Most of the directions of that research are focused on so-called smart polymeric materials which feature such basic functionalities as perception of changes, processing the obtained information, and responding to these changes. Smart materials are responsive to external stimuli, e.g., magnetic fields, significantly changing of their properties to obtain the desired and effective response to these stimuli. Such materials are currently used in almost every branch of science and technology. Magnetic particles contained in polymer, in order to achieve large magnetic forces, are aligned with the direction of the external magnetic field. Intermolecular attractive forces between particles result in their aggregation in complex networks which shortens the distance between them, and thereby increases the rigidity of the material. Mechanical properties can be modulated by magnetic field. Polymer composites feature new or improved physical and chemical properties. In particular, their water absorption capacity is reduced and their chemical resistance to acids and bases and frost resistance are increased as well. Due to their unique properties these materials can be widely used in the space industry, electrical engineering field, or automotive industry. Also, magnetorheological abrasive polishing of objects with complex shapes by an application of a constant magnetic field is a promising method of surface treatment of machine components. This method can be also applied to objects of complex shape using abrasive masses based on polymers and abrasive grains with ferromagnetic properties.

The original aspect of the present study is the application of a constant magnetic field with additional contribution of carbonyl iron during a polymerization of polymer composites and use of admixtures with specific properties. The constant magnetic field tended to increase the hardness and tensile strength of polymer composites or to increase their frost resistance. The admixture of expanded graphite increased the resistance of composites to corrosion, high temperatures, and electromagnetic radiation. The admixture of birch bark with a high cellulose content, containing betulin, results in anticancer, antiviral, and anti-inflammatory properties while the admixture of yellow dextrin improved the thickening and stiffening features of the composites.

## 2. Materials and Methods

### 2.1. Components of Polymeric Composites

**Figure 1:**
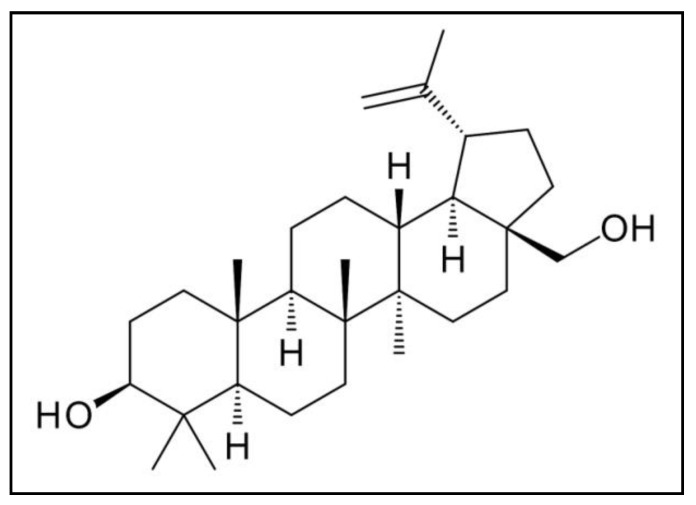
The structural formula of betulin present in birch bark.

The following components were used to prepare the polymer composites: Epidian 5 epoxy resin (Organika-Sarzyna, Nowa Sarzyna, Poland); IDA hardener (Organika-Sarzyna, Nowa Sarzyna, Poland); expanded graphite EG 290 with high resistance to corrosion, elevated temperature, electromagnetic radiation and compression; powder graphite 390 (BIOMUS company, Lublin, Poland, 7, Chemiczna St.); 100% birch bark, dried, with a high content of cellulose, containing betulin (Figure 1) with anti-cancer, antiviral (HIV-1), anti-inflammatory, and anti-allergic properties (DARY PODLASIA company, Bielsk Podlaski (Poland), 87a/3, Ogrodowa St.).

The following components were used to prepare the polymer composites: Epidian 5 epoxy resin (Organika-Sarzyna, Nowa Sarzyna, Poland), density 1.18–1.19 g/cm^3^, viscosity 20,000–30,000 mPa s, molar mass 700; IDA hardener (Organika-Sarzyna, Nowa Sarzyna, Poland), density 1.01–1.03 g/cm^3^, viscosity 150–300 mPa s; expanded graphite EG 290 with high resistance to corrosion, elevated temperature, electromagnetic radiation and compression, granulation 300–400 µm; powder graphite 390 (BIOMUS company, Lublin, Poland, 7, Chemiczna St.); and 100% birch bark, dried, with a high content of cellulose, containing betulin (Figure 1) with anti-cancer, antiviral (HIV-1), anti-inflammatory, and anti-allergic properties (DARY PODLASIA company, Bielsk Podlaski (Poland), 87a/3, Ogrodowa St.). Polymer composites on Epidian 5 epoxy resin matrix additionally contained magnetic particles with ferromagnetic properties in the form of carbonyl iron in the amount of 10% by weight, derived from the decomposition of iron pentacarbonyl at high temperature (97%, Alfa Aesar, Thermo Fisher Scientific, Winsford, UK), which allowed for the change of some properties of the composites in a constant magnetic field. The polymer composites also contained yellow pure dextrin (C_6_H_10_O_5_)_n_, where *n* is a number of molecules, with thickening, filling, and stiffening properties (BIOMUS company, Lublin, Poland, 7, Chemiczna St.), 99.5% content, density 2.8–3.5 g/cm^3^, granulation 5 µm.

### 2.2. Preparation of Test Samples

All tested samples were prepared according to the same procedure. After the composition of the sample was developed, the individual components were weighed and added in the following order: Epidian 5 epoxy resin, magnetic particles in the form of carbonyl iron (10% by wt.), and a filler (powder graphite 390, expanded graphite EG 290, birch bark with betulin or yellow dextrin, 20% by wt. and the IDA hardener for epoxy resin). The whole was mixed using a mechanical stirrer at a speed of 300 rpm for 180 s. The mixed liquid mass of the sample was placed in molders prepared in accordance with the PN-EN ISO 10210:2018-1 standard. Prior to the commencement of polymerization, the samples were divided into two groups. The first group of samples was placed between the poles of the laboratory electromagnet in a constant magnetic field with magnetic induction B = 0.5 T (samples PR1′–PR9′, PR17) and B = 0.25, 0.75, and 1.0 T (samples PR10–PR15), in which the polymerization of the matrix took place. The second group of samples (samples PR1–PR9, PR16) was placed outside the constant magnetic field (B = 0.0 T) during the polymerization of the matrix. The composition of the test samples and the magnetic induction used during the polymerization of the matrix are presented in Table 1.

### 2.3. Testing Methodology

#### 2.3.1. The Mechanical Tensile Strength

The mechanical tensile strength was tested using the Zwick/Roell Z050, KL 0.05 mechanical strength measuring machine with a measuring head of 50 kN, a test speed of 50 mm/min, and a tensile module speed of 5 mm/min (Zwick Roell, Ulm, Germany). The test speed is determined by the requirements specific to the standards for such types of composite materials (in contrast to classical thermoplastics). In addition, based on our experience with curable plastics, testing samples at lower speeds allows a more accurate analysis of processes at the polymer-filler interface. The impact tensile strength was tested according to the PN-EN ISO 8256:2006 standard. The test specimens were in the form of type 4 fittings with the following dimensions: length of 60 mm, width of 10 mm, measuring narrowing of 3 mm, free length between the handles of 25 mm, and radius of curvature of 20 mm. The static tensile strength was tested according to the PN-EN ISO 527:1998 standard. The test specimens were in the form of B1-type fittings with the following dimensions: length of 150 mm, width of 20 mm, measuring narrowing of 10 mm, free length between the handles of 115 mm, and radius of curvature of 60 mm. Impact strength is a measure of the brittleness of materials. It is determined by the work required to dynamically fracture the specimen in the longitudinal direction related to the cross-sectional area at the fracture site. The pendulum strikes a test specimen fixed on one side to a fixed holder with a certain amount of energy. As a result of the impact tension, the measurement registers the energy absorbed during the operation of the device, giving it numerically in kJ. The tensile test consists in uniaxial deformation of properly prepared samples and the measurement of the forces generated. This study is one of the basic sources of information on the mechanical properties of plastics. The quantities measured in this test are deformation (elongation) and deformation force. The absolute elongation is the difference between the final and the initial length of the sample. The tensile strength is therefore the maximum stress that the material transmits during short-term static stretching.

#### 2.3.2. The Hardness

The hardness was tested with a ball-shaped indenter in accordance with the method described in the PN-EN ISO 868:2004 standard using a Shore durometer (AFFRI Inc., Wood Dale, IL, USA). The concept of hardness in relation to plastics is defined by the resistance of the material when a suitable indenter is pressed vertically into its surface by applying a pressure sufficient to a permanent deformation of a specimen. In this method a steel ball is slowly pressing into the tested material. After a specified period of 60 s, the equilibrium state is established in which the increasing surface of the impression balances the load exerted by the indenting ball. The measure of hardness is the ratio of the loading force to the depth of the indentation in the tested material.

#### 2.3.3. The Surface Water Contact Angle and the Free Surface Energy

The surface water contact angle and the free surface energy were determined using the DSA 25E goniometer from KRUSS GmbH, Hamburg (Germany). The wettability of the surfaces was measured in the laboratory atmosphere at a relative humidity of 45 ± 5% and temperature of 22 ± 2 °C using a DSA-25 Drop Shape Analysis System (KROSS GmbH). Three liquids, namely, deionized water (POCH S.A., Gliwice, Poland), glycerine (POCH S.A., Gliwice, Poland), and diiodomethane droplets (POCH S.A., Gliwice, Poland) were used in the contact angle measurements. Drops with a volume of 2 µL were deposited on the surfaces using an automatic syringe. The static contact angle values were reported for five distinct drops deposited on each sample. The surface free energy was calculated using the Van Oss-Chaudhury-Good method.

#### 2.3.4. The Surface Topography and Roughness

The topography and roughness of composite surfaces were investigated with the Leica DCM8 optical profilometer (Leica Microsystems GmbH, Wetzlar, Germany). 

#### 2.3.5. The Water Absorption

The water absorption of the samples was tested as follows. The samples were cut into smaller fragments, described, and weighed on an analytical balance. The samples were then placed in a glass crystallizer filled with deionized water in such a way that they were completely submerged and left for 24 h. After 24 h, the samples were removed from the deionized water, dried at room temperature for 30 min, and weighed. The water absorption *n_w_* of the sample was calculated from the following Equation (1):

(1) $$n_{w}=\frac{m_{n}-m}{m}\;\cdot 100\%$$

where *m_n_* is the mass of the sample saturated with distilled water (in g) and *m* is the mass of the dry sample (in g). 

#### 2.3.6. The Frost Resistance

The frost resistance of the samples was tested as follows. The samples dried to a constant mass were immersed completely in distilled water for 24 h. After removing the sample from distilled water, it was placed on tissue paper for 30 min to dry and then placed in the freezer (−20 °C) for 24 h. After removal from the freezer, the samples were again immersed completely in distilled water for 24 h, then removed from the water, air-dried for 48 h, and weighed. The percentage of damage of sample S was defined as the relative loss of its mass calculated from the following Equation (2):

(2) $$S=\frac{m_{1}-m_{2}}{m_{1}}\cdot 100\%$$

where *m*_1_ is the mass of the dried test sample (in g) and *m*_2_ is the mass of the sample dried at the end of the test (in g). 

There are many different methods of testing frost resistance. The methods for determining resistance to freezing and thawing are included in the international standards PN-EN 772-18:2011, MON-EN 771-2:2011 and MON-EN 1338:2005. The frost resistance tests presented in this paper are based on the PN-EN 206 + Al:2016 standard and the national supplement PN-88/B-06250, which describes the so-called normal frost resistance test, based on the study of mass lost as a result of repeated freezing and thawing of samples. In our paper, frost resistance is defined as the percentage of weight loss of sample S due to damages, such as cracks and chips, caused by water freezing in this sample. Thus, the smaller the loss of the sample mass, the smaller the percentage of the sample damage and the greater its frost resistance.

The measurements of all parameters were performed five times for each sample, and the results presented in the graphs are arithmetic mean values from five measurements. Errors in the measurements of water absorption, frost resistance, and mechanical strength were ±0.003%, ±0.002%, and ±0.1 MPa, respectively.

#### 2.3.7. Constant Magnetic Field

A constant magnetic field with magnetic induction B of 0.25, 0.5, 0.75, and 1.0 T was generated using a laboratory electromagnet model LS-EM-7V with LS-648 control power supply, and magnetic induction was measured using the LS-F41-FC teslameter and the LS-FP-2X-250-TF15 Hall probe (all equipment manufactured by Lake Shore Cryotronics, Westerville, OH, USA).

## 3. Results and Discussion

### 3.1. Testing of Mechanical Properties

#### 3.1.1. Hardness

**Figure 2:**
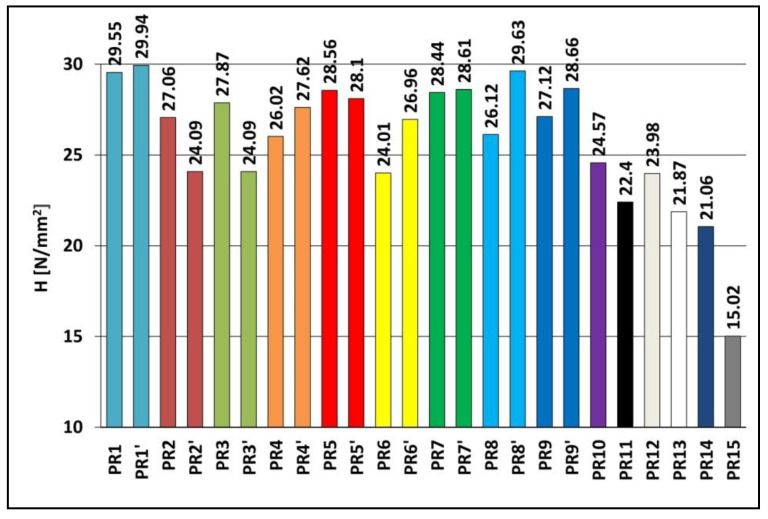
Hardness H of Epidian 5 epoxy resin and polymer composites on the Epidian 5 epoxy resin matrix obtained in a constant magnetic field environment (PR1′–PR9′ and PR10–PR15 samples) or without a constant magnetic field (PR1–PR9 samples). Sample composition is presented in Table 1.

The hardness of the PR1–PR9, PR1′–PR9′ and PR10–PR15 samples was tested by the ball indenter pressing method in accordance with PN-EN ISO 868:2004. Hardness measurement results are shown in Figure 2. 

Polymerization of Epidian 5 resin, which is the matrix of the composite, alone, in a constant magnetic field, caused an increase in hardness from 27.12 N/mm^2^ (sample PR9, B = 0.0 T) to 28.66 N/mm^2^ (sample PR9′, B = 0.5 T); composite with an admixture of powder graphite caused an increase from 29.55 N/mm^2^ (sample PR1, B = 0.0 T) to 29.94 N/mm^2^ (sample PR1′, B = 0.5 T) and composite with yellow dextrin caused an increase from 28.44 N/mm^2^ (sample PR7, B = 0.0 T) to 28.61 N/mm^2^ (sample PR7′, B = 0.5 T), while a decrease in the hardness of the composite resulted from an admixture of expanded graphite, from 27.06 N/mm^2^ (sample PR2, B = 0.0 T) to 24.09 N/mm^2^ (sample PR2′, B = 0.5 T) and birch bark composite with betulin, from 28.56 N/mm^2^ (sample PR5, B = 0.0 T) to 28.10 N/mm^2^ (sample PR5′, B = 0.5 T). After the introduction of carbonyl iron admixture and polymerization in a constant magnetic field, there was an increase in the hardness of the expanded graphite composite from 26.02 N/mm^2^ (sample PR4, B = 0.0 T) to 27.62 N/mm^2^ (sample PR4′, B = 0.5 T), a composite with an admixture of birch bark with betulin, produced a similar increase, from 24.01 N/mm^2^ (sample PR6, B = 0.0 T) to 26.96 N/mm^2^ (sample PR6′, B = 0.5 T), and yellow dextrin composite, from 26.12 N/mm^2^ (sample PR8, B = 0.0 T) to 29.93 N/mm^2^ (sample PR8′, B = 0.5 T), while there was a decrease in the hardness of the powder graphite composite, declining from 27.87 N/mm^2^ (PR3 sample, B = 0.0 T) to 24.09 N/mm^2^ (PR3′ sample, B = 0.5 T).

**Figure 3:**
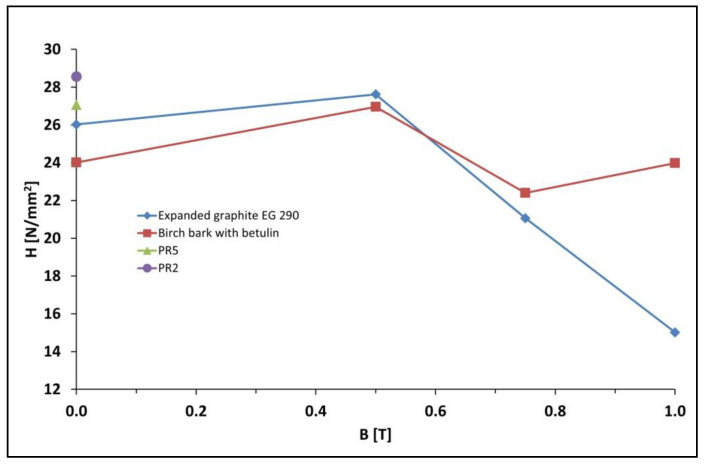
Hardness H of polymer composites on Epidian 5 epoxy resin matrix with the addition of magnetic particles in the form of carbonyl iron and an admixture of expanded graphite (samples PR4, PR4′, PR14 and PR15), or an admixture of birch bark with betulin (samples PR6, PR6′, PR11 and PR12) from magnetic induction B used during matrix polymerization. For comparison, the hardness of PR2 and PR5 composites without carbonyl iron is also given. The composition of polymer composites is presented in Table 1.

The relationship between the hardness of polymer composites on Epidian 5 epoxy resin matrix with the addition of magnetic particles in the form of carbonyl iron, and with an admixture of expanded graphite (samples PR4, PR4′, PR14 and PR15), or with an admixture of birch bark with betulin (samples PR6, PR6′, PR11 and PR12) and the induction of a constant magnetic field used during matrix polymerization (Figure 3) was investigated. 

The introduction of carbonyl iron into polymer composites on the Epidian 5 epoxy resin matrix with an admixture of expanded graphite (sample PR4) or with an admixture of birch bark with betulin (sample PR6) obtained without a constant magnetic field resulted in a decrease in their hardness to 20.62 and 24.01 N/mm^2^ respectively, compared to composites without the addition of carbonyl iron, i.e., samples PR2 (H = 27.06 N/mm^2^) and PR5 samples (H = 28.56 N/mm^2^). When the polymerization of PR4′ and PR6′ composites took place in a constant magnetic field with induction B = 0.5 T, their hardness increased to 27.62 and 26.96 N/mm^2^, respectively. However, a further increase in magnetic field induction caused a decrease in the hardness of the composites, and the lowest hardness was found in the case of composites PR12 (H = 23.98 N/mm^2^) and PR15 (H = 15.02 N/mm^2^), both obtained with high magnetic field induction B = 1.0 T, causing the rigidity of the material, and consequently its brittleness.

#### 3.1.2. Impact Tensile Strength

**Figure 4:**
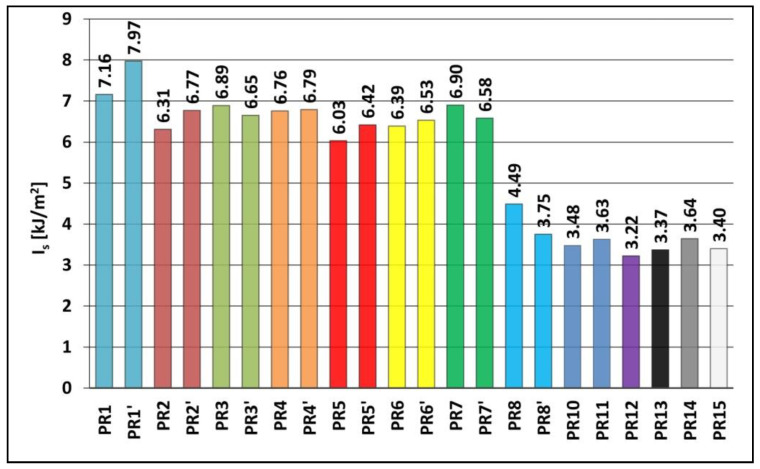
Impact tensile strength I_s_ of polymer composites on Epidian 5 epoxy resin matrix obtained in a constant magnetic field environment (PR1′–PR8′ and PR10–PR15 samples) or without a constant magnetic field (PR1–PR8 samples). Sample composition is presented in Table 1.

**Figure 5:**
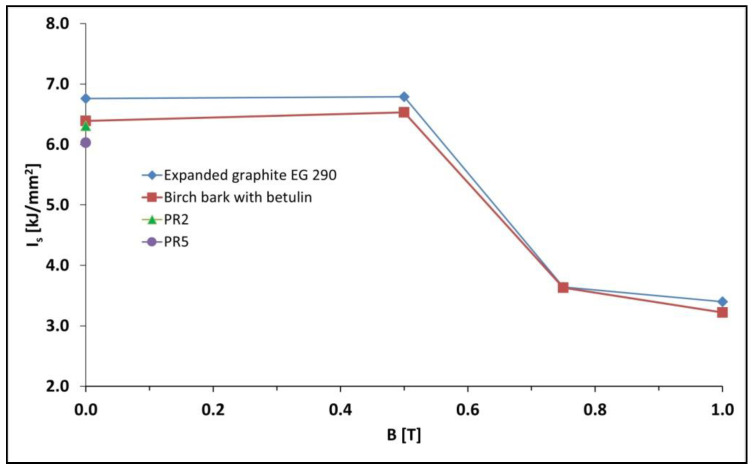
Diagram of the dependence of the impact tensile strength I_s_ of polymer composites on Epidian 5 epoxy resin matrix with the addition of magnetic particles in the form of carbonyl iron and with an admixture of expanded graphite (samples PR4, PR4′, PR14, and PR15) or with an admixture of birch bark with betulin (samples PR6, PR6′, PR11, and PR12) from magnetic induction B used during matrix polymerization. For comparison, the impact tensile strengths of PR2 and PR5 composites without carbonyl iron are also given. The composition of polymer composites is presented in Table 1.

The impact tensile strengths I_s_ of the PR1–PR8, PR1′–PR8′ and PR10–PR15 samples were tested in accordance with the EN ISO 8256:2006 standard. The results of the measurements are presented in Figure 4. For polymer composites with an admixture of expanded graphite and birch bark containing betulin, a graph of the dependence of the impact tensile strength I_s_ on magnetic induction B was plotted (Figure 5).

Polymerization in a constant magnetic field resulted in an increase in the impact tensile strength of the powder graphite composite from 7.16 kJ/m^2^ (sample PR1, B = 0.0 T) to 7.97 kJ/m^2^ (sample PR1′, B = 0.5 T), expanded graphite composite resulted in an increase from 6.31 kJ/m^2^ (sample PR2, B = 0.0 T) to 6.77 kJ/m^2^ (sample PR2′, B = 0.5 T), and birch bark composite with betulin, from 6.03 kJ/m^2^ (sample PR5, B = 0.0 T) to 6.42 kJ/m^2^ (sample PR5′, B = 0.5 T) and a decrease resulted in the tensile strength of the yellow dextrin composite from 6.90 kJ/m^2^ (sample PR7, B = 0.0 T) to 6.58 kJ/m^2^ (sample PR7′, B = 0.5 T). The introduction of carbonyl iron and polymerization in a constant magnetic field causes a decrease in the impact tensile strength of the powder graphite composite from 6.89 kJ/m^2^ (sample PR3, B = 0.0 T) to 6.65 kJ/m^2^ (sample PR3′, B = 0.5 T) and yellow dextrin composite causes a decrease from 4.49 kJ/m^2^ (sample PR8, B = 0.0 T) to 3.75 kJ/m^2^ (sample PR8′, B = 0.5 T) and increase in the impact tensile strength of the expanded graphite composite is seen from 6.76 kJ/m2 (sample PR4, B = 0.0 T) to 4.79 kJ/m^2^ (sample PR4′, B = 0.5 T) and birch bark composite with betulin, from 6.39 kJ/m^2^ (sample PR6, B = 0.0 T) to 6.53 kJ/m^2^ (sample PR6′, B = 0.5 T).

The introduction of carbonyl iron into polymer composites on Epidian 5 epoxy resin matrix with an admixture of expanded graphite (sample PR4) or with an admixture of birch bark with betulin (sample PR6) obtained without a constant magnetic field resulted in an increase in impact tensile strength to, respectively, 6.76 and 6.39 kJ/mm^2^, compared with composites without the addition of carbonyl iron, i.e., sample PR2 (I_s_ = 6.31 kJ/mm^2^) and sample PR5 (I_s_ = 6.03 kJ/mm^2^). When the polymerization of composites took place in a constant magnetic field with induction B = 0.5 T, there was a slight increase in their impact tensile strength to 6.79 kJ/mm^2^ (sample PR4′) and 6.53 kJ/mm^2^ (sample PR6′). However, a further increase in magnetic field induction caused a decrease in the impact tensile strength of the composites, and the lowest strength was demonstrated by the PR12 (Is = 3.22 kJ/mm^2^) and PR15 composites (Is = 3.40 kJ/mm^2^) obtained with high magnetic field induction B = 1.0 T causing rigidity of the material, and thus its brittleness.

#### 3.1.3. Static Tensile Strength

**Figure 6:**
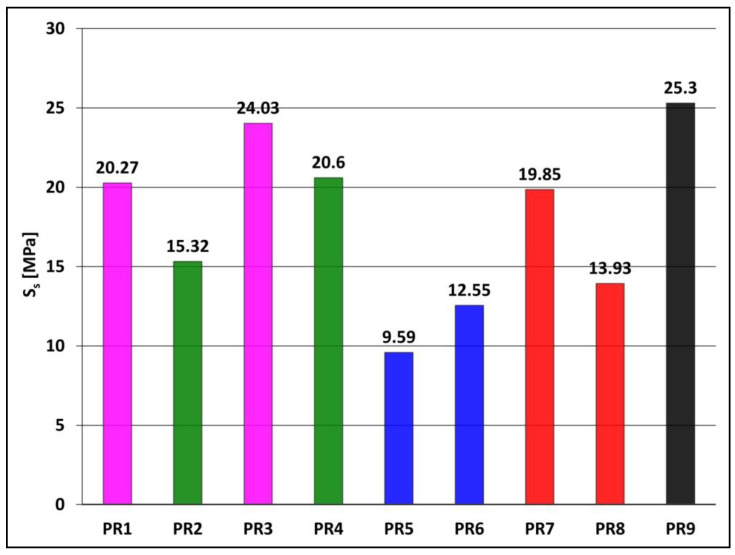
Static tensile strength S_s_ of Epidian 5 epoxy resin (sample PR9) and polymer composites on Epidian 5 epoxy resin matrix (samples PR1–PR8), obtained without the action of a constant magnetic field (B = 0.0 T). Sample composition is presented in Table 1.

The static tensile strength S_s_ of samples PR1-PR9, which were obtained without a constant magnetic field, was tested in accordance with EN ISO 527-2. The results of static tensile strength tests are presented in Figure 6. 

The highest static tensile strength was shown by a sample made of Epidian 5 resin alone (sample PR9, S_s_ = 25.3 MPa), and all tested PR1-PR8 composites had a lower tensile strength of 9.59 to 24.03 MPa. The introduction of carbonyl iron into composites with powder graphite (sample PR1, S_s_ = 20.27 MPa), with expanded graphite (sample PR2, S_s_ = 15.32 MPa), or with birch bark with betulin (sample PR5, S_s_ = 9.59 MPa) results in an increase in static tensile strength to 24.03 MPa (sample PR3), 20.60 MPa (sample PR4) and 12.55 MPa (sample PR6), respectively. However, the introduction of carbonyl iron into a composite with yellow dextrin (sample PR7, S_s_ = 19.85 MPa) causes a decrease in static tensile strength to 13.93 MPa (sample PR8).

### 3.2. Water Absorption and Frost Resistance

**Figure 7:**
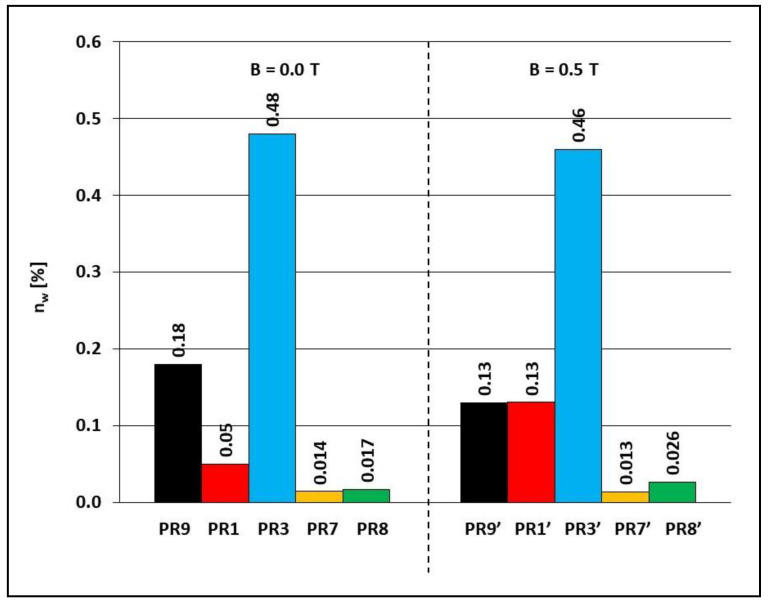
Water absorption *n_in_* Epidian 5 epoxy resin and polymer composites on the Epidian 5 epoxy resin matrix obtained in a constant magnetic field environment (B = 0.5 T, samples PR1′, PR3′, PR7′, PR8′ and PR9′) or without the action of a constant magnetic field (B = 0.0 T, samples PR1, PR3, PR7, PR8 and PR9). Sample composition is presented in Table 1.

**Figure 8:**
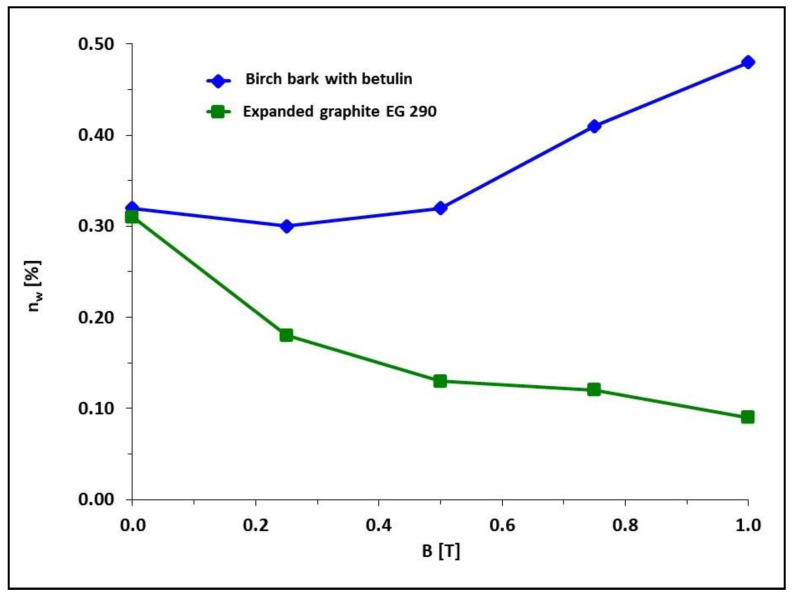
Dependence of water absorption *n_w_* of polymer composites on Epidian 5 matrix with selected birch bark additives with betulin (samples PR5, PR5′, PR10, PR11, and PR12) or expanded graphite (samples PR2, PR2′, PR13, PR14, and PR15) and admixture to each composite of magnetic particles of carbonyl iron, on magnetic induction B. Sample composition is presented in Table 1.

For water absorption and frost resistance tests, samples PR1, PR3, PR7, PR8, and PR9 obtained in the presence of a constant magnetic field with induction B = 0.5 T and samples PR1′, PR3′, PR7′, PR8′, and PR9′, obtained without the presence of a constant magnetic field, were selected. Water absorption determines the ability of a particular material to absorb water, and in the case of this work, by a polymer composite, and determines the maximum water saturation of a given material. The results of water absorption tests are presented in Figure 7 and Figure 8. 

When the polymerization of the Epidian 5 epoxy resin alone was carried out without the presence of a constant magnetic field (sample PR9), it had a water absorption of 0.18%. Subsequently, powdered graphite, yellow dextrin, or yellow dextrin with carbonyl iron were added to it, and there was a decrease in water absorption to, respectively, 0.05% (sample PR1), 0.014% (sample PR7), and 0.017% (sample PR8), while the introduction of powder graphite and carbonyl iron resulted in an increase in water absorption to 0.48% (sample PR3). When the polymerization of Epidian 5 epoxy resin was carried out in the presence of a constant magnetic field with induction B = 0.5 T, a decrease in water absorption of the resin alone from 0.18% (sample PR9) to 0.13% (sample PR9′), in the composite with powder graphite and with carbonyl iron from 0.48% (sample PR3) to 0.46% (sample PR3′) and in the composite with yellow dextrin from 0.014% (sample PR7) to 0.013% (sample PR7′) was found, while an increase in water absorption was observed in the case of the composite with powder graphite content, from 0.05% (sample PR1) to 0.13% (sample PR1′) and a composite with yellow dextrin and carbonyl iron from 0.017% (sample PR8) to 0.026% (sample PR8).

In the case of a polymer composite on Epidian 5 epoxy resin matrix with an admixture of expanded graphite and with carbonyl iron (samples PR2, PR2′, PR13, PR14, PR15), an increase in magnetic field induction during polymerization resulted in a decrease in water absorption, and the lowest absorbability of 0.09% was achieved at B = 1.0 T (sample PR15). In contrast, when the Epidian 5 epoxy resin matrix composite with carbonyl iron and birch bark with betulin polymerized in a constant magnetic field, an increase in magnetic field induction resulted in an increase in the absorbability of the composite with the maximum value of 0.48% for B = 1.0 T (sample PR12). 

**Figure 9:**
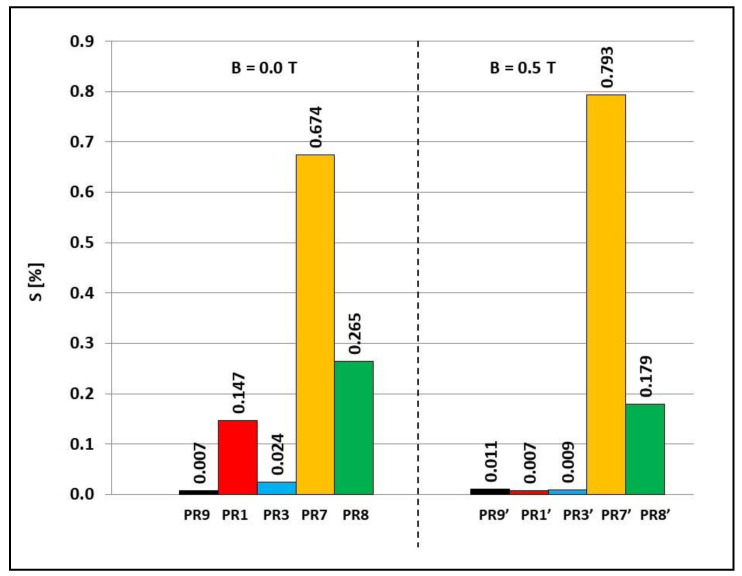
Percentage of damage to Epidian 5 epoxy resin sample S and polymer composites on Epidian 5 epoxy resin matrix obtained in a constant magnetic field environment (B = 0.5 T, samples PR1′, PR3′, PR7′, PR8′ and PR9′) or without a constant magnetic field (B = 0.0 T, samples PR1, PR3, PR7, PR8 and PR9). Sample composition is presented in Table 1.

**Figure 10:**
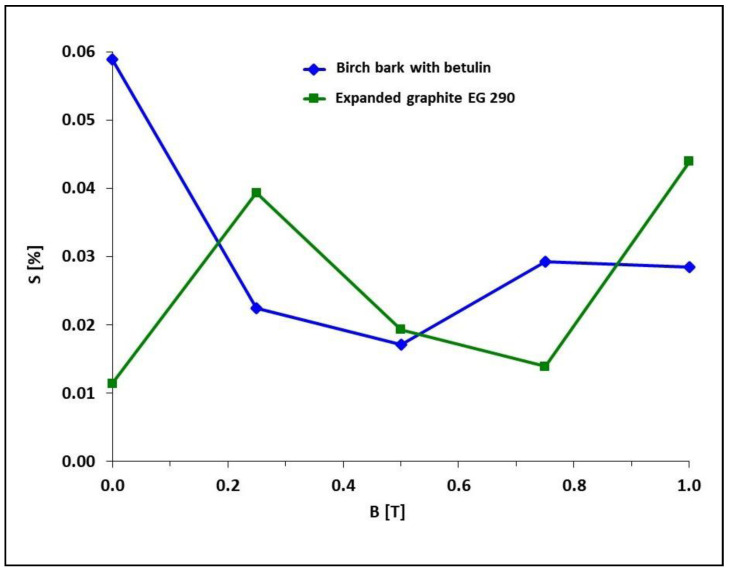
The dependence of the percentage of damage to sample S of polymer composites on Epidian 5 epoxy resin matrix with an admixture of magnetic particles of carbonyl iron with the addition of birch bark with betulin (PR5, PR5′, PR10, PR11, PR12), or expanded graphite (PR2, PR2′, PR13, PR14, PR15) on the magnetic field induction B. The composition of the samples is presented in Table 1.

The results of the frost resistance tests of the composites are presented in Figure 9 and Figure 10. The exposure to negative temperature resulted in an increase in the volume of water absorbed by the composites during the transition to the solid state. Cracking of the samples was a negative effect of water crystallization during freezing of the composites.

The frost resistance of all tested composites obtained without the presence of a constant magnetic field was reduced by the addition to Epidian 5 epoxy resin matrix (sample PR9, S = 0.007%); powder graphite (sample PR1), powder graphite and carbonyl iron (sample PR3), yellow dextrin (sample PR7), and yellow dextrin and carbonyl iron (sample PR8), demonstrated reduced frost resistance, because their percentage of damage to sample S increased to 0.147%, 0.024%, 0.674%, and 0.265%, respectively. Polymerization in a constant magnetic field also caused a decrease in the frost resistance of the Epidian 5 epoxy resin matrix (sample PR9′) and composite with the addition of yellow dextrin (sample PR7′), since their percentage of damage to sample S increased, respectively, from 0.007% and 0.674% to 0.011% and 0.793%. On the other hand, polymerization in a constant magnetic field of the composite with powder graphite (sample PR1′), the composite with powder graphite and carbonyl iron added (sample PR3′), and the composite with the addition of yellow dextrin and carbonyl iron (sample PR8′) caused an increase in frost resistance, since their percentage of damage to sample S decreased from 0.147%, 0.024%, and 0.265% to 0.007%, 0.009%, and 0.179%, respectively.

The effect of induction of a constant magnetic field on frost resistance of the composites with carbonyl iron particles with the addition of botulin-containing birch bark and with the addition of expanded graphite is not the same. In the case of the composite with carbonyl iron particles and birch bark added, an increase in magnetic field induction to 0.25 and 0.5 T causes an increase in frost resistance, i.e., a decrease in the percentage of damage to sample S respectively to 0.022% (sample PR10) and 0.017% (sample PR6). On the other hand, a further increase in magnetic field induction to 0.75 and 1.0 T causes a decrease in the frost resistance of the composites, as their percentage of damage to sample S increases, respectively, to 0.029% (sample PR11) and 0.028% (sample PR12). Similarly, in the case of the composite with carbonyl iron particles and expanded graphite, an increase in magnetic field induction to 0.25 T causes an increase in the percentage of damage to sample S to 0.039% (sample PR13), i.e., a decrease in frost resistance, then an increase in frost resistance is observed with the magnetic field induction of 0.5 and 0.75 T, i.e., a decrease in the percentage of damage to sample S to, respectively, 0.019% (sample PR4′) and 0.014% (sample PR14), and again a decrease in frost resistance at B = 1.0 T (sample PR15, S = 0.044%).

### 3.3. Surface Water Contact Angle and Free Surface Energy

Surface water contact angle θ is the angle formed between the flat surface of a solid body and the plane tangential to the surface of the liquid bordering that solid. In particular, the water contact angle of the surface θ is a measure of the hydrophilic/hydrophobic properties of this surface, namely, if θ < 90° then the wettability of the surface by water is high (hydrophilic surface), if θ > 90° then the wettability of the surface by water is low (hydrophobic surface).

**Figure 11:**
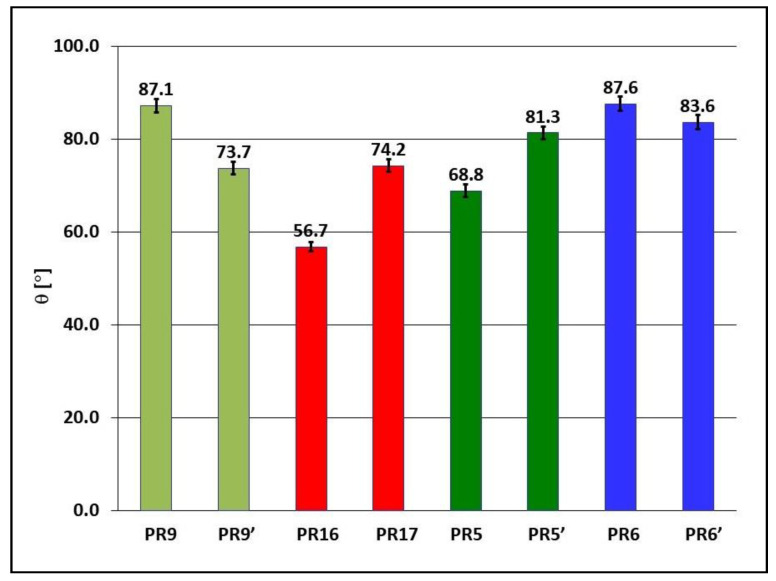
The surface water contact angle θ of Epidian 5 epoxy resin and polymer composites on Epidian 5 epoxy resin matrix obtained in an environment of constant magnetic field (B = 0.5 T, samples PR5′, PR6′, PR9,′ and PR17) or without the action of a constant magnetic field (B = 0.0 T, samples PR5, PR6, PR9 and PR16). Sample composition is presented in Table 1.

In order to determine the hydrophilic/hydrophobic properties of the PR9, PR9′, PR16, PR17, PR5, PR5′, PR6, and PR6′ samples, the surface water contact angle θ was determined. The results of measurements of the angle of the surface contact with water θ are presented in Figure 11.

Both the Epidian 5 epoxy resin alone (samples PR9 and PR9′) and all composites on its matrix obtained both in the presence of a magnetic field (samples PR17, PR5′, and PR6′) and without the presence of a magnetic field (samples PR16, PR5, and PR6), show high wettability surface wettability by water (high hydrophilicity), because the surface water contact angle is θ < 90°. Polymerization of Epidian 5 epoxy resin alone in a constant magnetic field increases the hydrophilicity of its surface, since the water contact angle θ is reduced from 87.1° (sample PR9) to 73.7° (sample PR9′). The admixture of carbonyl iron to the matrix of Epidian 5 epoxy resin polymerizing without the presence of a constant magnetic field (sample PR16) significantly increases the hydrophilic surface of the composite (θ = 56.7°). However, when the polymerization of this composite takes place in the presence of a constant magnetic field (sample PR17), the hydrophilicity of its surface decreases (θ = 74.2°).

The admixture of birch bark with betulin to the Epidian 5 epoxy resin matrix polymerizing without the presence of a constant magnetic field (sample PR5) causes an increase in the hydrophilicity of the composite surface (θ = 68.8°), compared to the hydrophilicity of the surface of Epidian 5 epoxy resin (sample PR9, θ = 87.1°), however, the presence of a constant magnetic field during polymerization of this composite (sample PR5′) reduces the hydrophilicity of its surface (θ = 81.3°). The addition of a mixture of magnetic carbonyl iron and birch bark to the Epidian 5 epoxy resin matrix polymerizing without the presence of a constant magnetic field (sample PR6) practically does not change the hydrophilicity of the composite (θ = 87.6°) compared to the hydrophilicity of the surface of Epidian 5 epoxy resin (sample PR9, θ = 87.1°). Polymerization of this composite in the presence of a constant magnetic field (sample PR6′) causes a slight increase in the hydrophilicity of its surface (θ = 83.6°).

**Figure 12:**
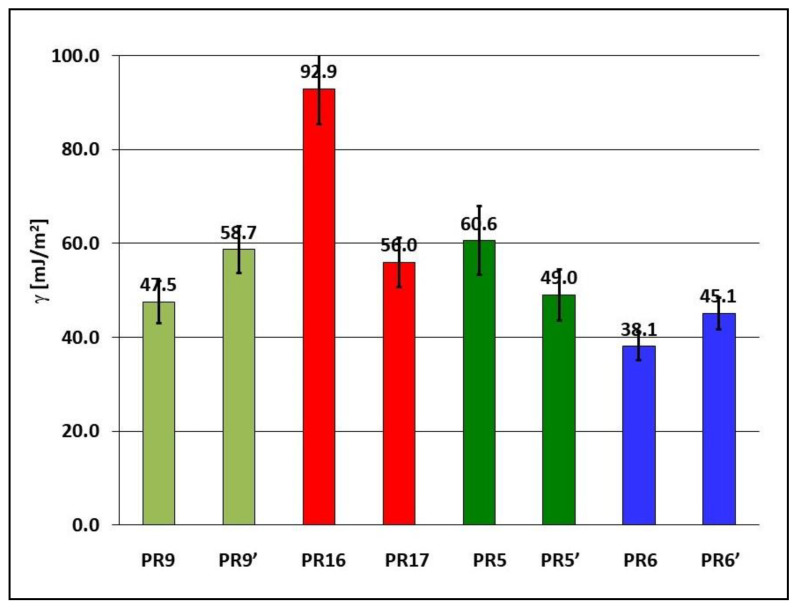
Free surface energy γ of Epidian 5 epoxy resin and polymer composites on Epidian 5 epoxy resin matrix obtained in a constant magnetic field environment (B = 0.5 T, samples PR5′, PR6′, PR9′, and PR17) or without the action of a constant magnetic field (B = 0.0 T, samples PR5, PR6, PR9, and PR16). Sample composition is presented in Table 1.

Free surface energy γ is one of the thermodynamic quantities describing the equilibrium state of atoms in the surface layer of a particular material. Free surface energy γ reflects a specific state of imbalance in intermolecular interactions present at the interface of the phases of two media. Free surface energy γ is equal to the work needed to form a surface area unit during the separation of two phases in the equilibrium state in a reversible isothermal process. The free surface energy of the PR9, PR9′, PR16, PR17, PR5, PR5′, PR6, and PR6′ samples was calculated from the determined angles of the surface contact with water, diiodomethane and glycerin. The results of the free surface energy calculations are presented in Figure 12. 

When polymerization of Epidian 5 epoxy resin took place without the presence of a constant magnetic field (sample PR9), its free surface energy was 47.5 mJ/mm^2^, and the introduction of carbonyl iron or birch bark with betulin as an admixture caused an increase in the free surface energy of the composites to 92.9 mJ/mm^2^ (sample PR16) and 60.6 mJ/mm^2^ (sample PR5), respectively. However, when carbonyl iron and birch bark with betulin were introduced as an admixture, the free surface energy of this composite was reduced to 38.1 mJ/mm^2^ (sample PR6). The presence of a constant magnetic field during the polymerization of Epidian 5 epoxy resin alone increased its free surface energy from 47.5 mJ/m^2^ (sample PR9, B = 0.0 T) to 58.7 mJ/m^2^ (sample PR9′, B = 0.5 T). The introduction of an admixture of carbonyl iron into the matrix of Epidian 5 epoxy resin polymerizing without the presence of a constant magnetic field significantly increased its free surface energy from 47.5 mJ/m^2^ (sample PR9) to 92.9 mJ/m^2^ (sample PR16). However, when the polymerization of this composite took place in a constant magnetic field (sample PR17, B = 0.5 T), its surface energy decreased to 56.0 mJ/mm^2^. The introduction of birch bark with betulin into Epidian 5 epoxy resin polymerizing without the presence of a constant magnetic field increased its free surface energy from 47.5 mJ/m^2^ (sample PR9) to 60.6 mJ/m^2^ (sample PR5). When polymerization took place in the presence of a constant magnetic field (sample PR5′), the free surface energy was reduced to 49.0 mJ/mm^2^. If a mixture of magnetic carbonyl iron and birch bark with betulin (in total approx. 30% by mass) was used as an admixture to Epidian 5 epoxy resin, and polymerization took place without a constant magnetic field, a reduction in the free surface energy of the composite to 38.1 mJ/mm^2^ was observed (sample PR6). When polymerization of this composite took place in the presence of a constant magnetic field (sample PR6′, B = 0.5 T), its free surface energy was increased to 45.1 mJ/mm^2^.

### 3.4. Surface Testing of Polymer Composites

The selected samples of polymer composites on Epidian 5 epoxy resin matrix with an admixture of carbonyl iron (samples PR16 and PR17), or with an admixture of birch bark and carbonyl iron (samples PR6 and PR6′) were subjected to microscopic measurements of their surface profiles and topography. The surface profile is characterized by the surface roughness parameters Rp, Rz, Rt, Ra, Rq, Rdq, and Rdc (the meaning of the symbols is given in Table 2). Measurements of the surface profile of the composites and its topography were performed with the use of a Leica DCM8 optical profilometer from Technolutions.

**Figure 13:**
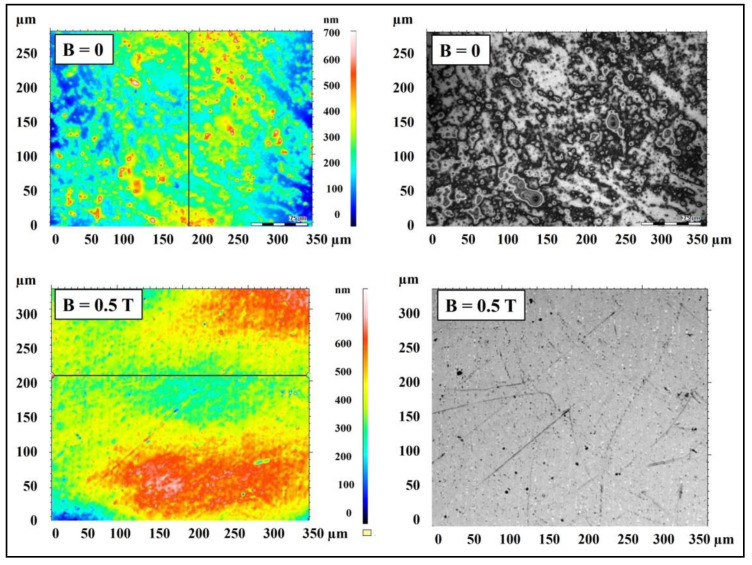
Confocal microscopy images of polymer composite on Epidian 5 epoxy resin matrix with an admixture of magnetic particles in the form of carbonyl iron (10% by weight) obtained without a constant magnetic field (B = 0.0 T, sample PR16) and in a constant magnetic field environment (B = 0.5 T, sample PR17).

Figure 13 presents microscopic images of polymer composites on an Epidian 5 epoxy resin matrix with an admixture of magnetic particles in the form of 10% by wt. carbonyl iron obtained in an environment of a constant magnetic field with an induction of B = 0.5 T (sample PR17) or without a constant magnetic field (sample PR16). 

The surface of the PR16 composite obtained without the presence of a constant magnetic field has many depressions and bulges formed by accumulated magnetic particles of carbonyl iron. On the other hand, the surface of the PR17 composite obtained in a constant magnetic field does not have such bulges, because the magnetic particles were absorbed into the material and were arranged along the force lines of the magnetic field.

**Figure 14:**
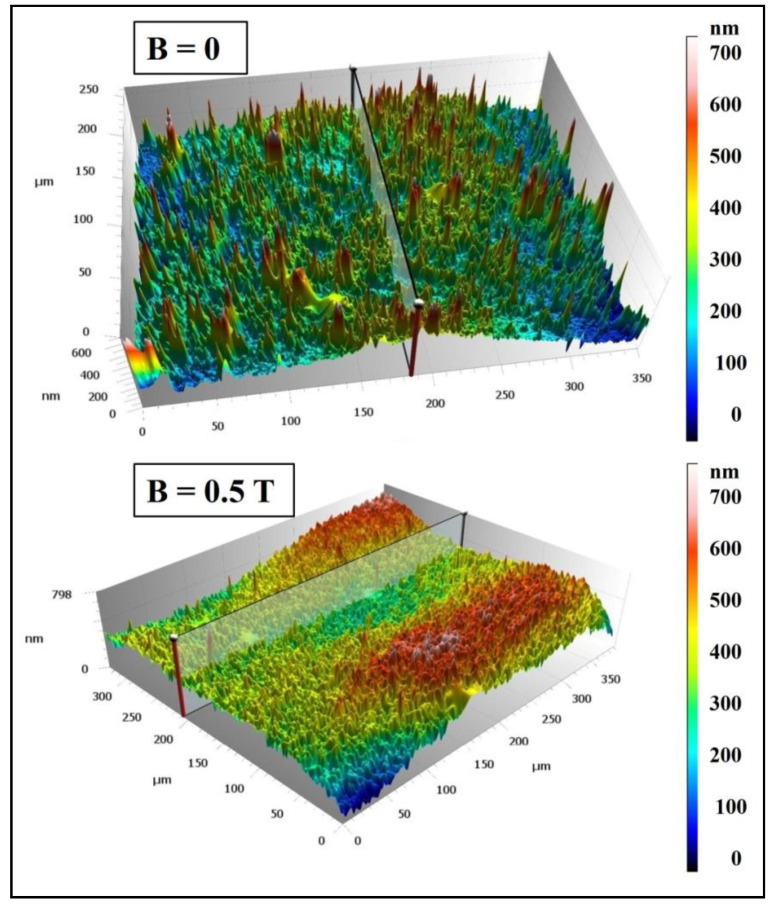
Microscopic images showing the surface topography of a polymer composite on Epidian 5 epoxy resin matrix with an admixture of carbonyl iron magnetic particles (10% by weight) obtained in a constant magnetic field environment (B = 0.5 T, sample PR17) and without a constant magnetic field (B = 0.0 T, sample PR16).

Figure 14 presents the surface topography of polymer composites on Epidian 5 epoxy resin matrix with an admixture of magnetic particles in the form of 10% by wt. carbonyl iron, obtained in a constant magnetic field environment with an induction of B = 0.5 T (sample PR17) and without a magnetic field (B = 0.0 T, sample PR16).

**Figure 15:**
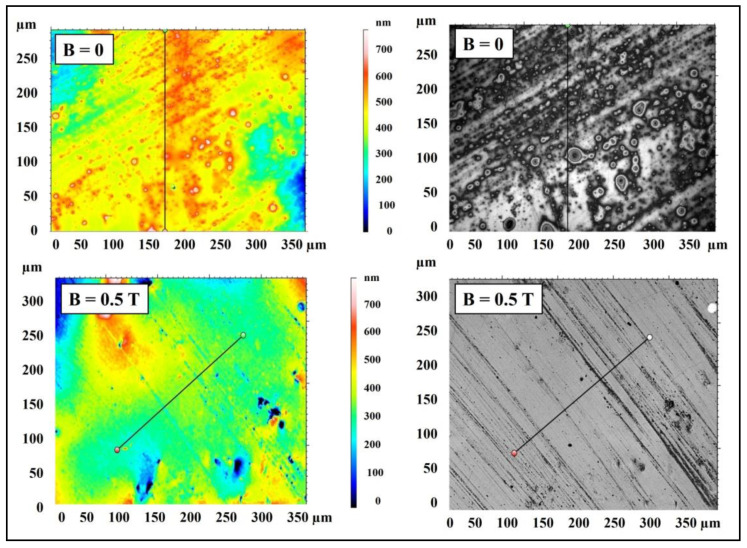
Confocal microscopy images of polymer composite on Epidian 5 epoxy resin matrix with the addition of magnetic particles of carbonyl iron (10% by weight) and birch bark with betulin (20% by weight) obtained in a constant magnetic field environment (B = 0.5 T, sample PR6′) and without the presence of a constant magnetic field (B = 0.0 T, sample PR6).

The surface of the PR17 composite obtained in a constant magnetic field is characterized by greater smoothness and greater compaction compared to the surface of the PR16 composite obtained without the presence of a constant magnetic field. Figure 15 shows microscopic images of polymer composites on Epidian 5 epoxy resin matrix with the addition of a mixture of magnetic particles of carbonyl iron (10% by wt.) and birch bark with betulin (20% by wt.) obtained in the presence of a constant magnetic field (B = 0.5 T, sample PR6′) and without the presence of a constant magnetic field (B = 0.0 T, sample PR6).

The surface of the PR6 composite obtained without the presence of a constant magnetic field has many bulges and depressions formed by accumulated magnetic particles of carbonyl iron, while the PR6′ composite obtained in a constant magnetic field with an induction of B = 0.5 T does not have such bulges, because they have been absorbed into the material and arranged along the force lines of the magnetic field. 

**Figure 16:**
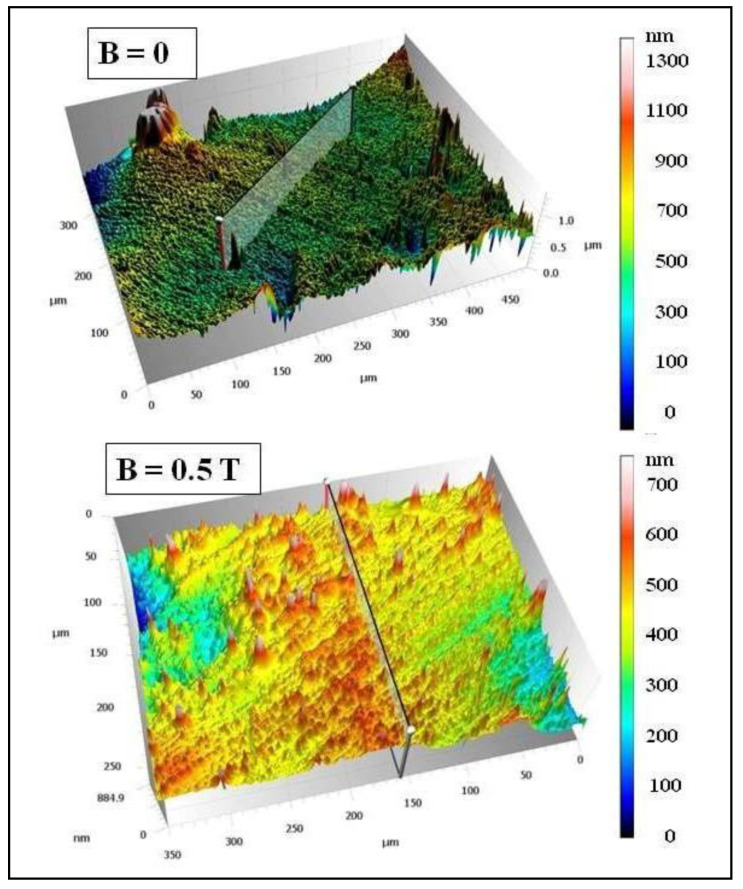
Microscopic images showing the surface topography of the polymer composite on Epidian 5 epoxy resin matrix with the addition of magnetic particles of carbonyl iron (10% by weight) and birch bark with betulin (20% by weight) obtained in a constant magnetic field environment (B = 0.5 T, sample PR6′) and without the participation of a constant magnetic field (B = 0.0 T, sample PR6).

The surface topography of the PR6 and PR6′ polymer composites is presented in Figure 16.

The surface of the PR6′ composite obtained in a constant magnetic field is characterized by greater smoothness and greater compaction compared to the surface of the PR6 composite obtained without the presence of a constant magnetic field. This is confirmed by the results of measurements of surface roughness of the composites obtained in a constant magnetic field with induction B = 0.5 T (samples PR17 and PR6′) and without the presence of a constant magnetic field (B = 0.0 T, samples PR16 and PR6), which are presented in Table 2.

Since all the determined surface roughness parameters are lower in the case of the PR17 and PR6′ composites obtained in the presence of a constant magnetic field than in the case of the PR16 and PR6 composites obtained without the presence of a constant magnetic field, it can be concluded that the surfaces of the PR17 and PR6′ composites have a greater smoothness than do the surfaces of the PR16 and PR6 composites.

### 3.5. General Remarks

The results of our investigations of epoxy resin-based composites with such fillers as expanded graphite, powder graphite, birch bark, and yellow dextrin presented in this paper are in line with the other published findings. In fact, epoxy composites reinforced with expanded graphite were found to display improved mechanical properties, such as elongation at break, load at break, tensile stress at break, and tensile strain at break [22,23,24]. The plywood panels bonded with urea-formaldehyde adhesive with birch bark as a filler showed satisfactory mechanical properties and a decrease in the release of formaldehyde [25,26]. The epoxy resin composites with carbon fibers as a reinforcement and powder graphite as a filler were also characterized as those with improved properties in tensile and flexural tests [27]. Finally, cement-based composites with dextrin filler have been proved as a construction material with increased compressive strength [28].

On the other hand, some of our results are surprising and cannot be explained, taking into account the bulk macroscopic properties. For example, the water absorption of composite PR3 (Figure 7), in which the epoxy resin matrix is hydrophilic due to the polar epoxy, hydroxyl, and amine groups, with hydrophobic powder graphite and carbonyl iron is higher than the neat epoxy resin (sample PR1). In turn, epoxy resin composite PR7 with hydrophilic yellow dextrine shows a lower water absorption than the neat epoxy resin (sample PR1). For comparison, the water absorption of epoxy resin-based biocomposites with expanded graphite was decreased with an increased amount of the hydrophobic filler [23]. In order to thoroughly investigate the relationship between internal structure of composites and their macroscopic properties, the scanning electron microscopy images of their cross-sections should be taken [29]. This could provide an insight into a such bulk phenomena as aggregation, dispersion, and orientation of microparticles which would allow researchers to estimate interactions at the microparticle/epoxy resin matrix interface. Such studies are planned by the authors in the future.

## 4. Conclusions

The paper presents the results of research on new polymer composites on the Epidian 5 epoxy resin matrix containing as admixtures (in the amount of 20% by weight) expanded graphite (with high corrosion resistance, heat resistance, radiation resistance), powder graphite, birch bark containing betulin (a compound with anti-cancer, antiviral, anti-inflammatory and anti-allergic effects) and yellow dextrin with thickening, filling and stiffening properties. Some of the composites additionally contained magnetic particles of carbonyl iron with ferromagnetic properties in the amount of 10% by weight, derived from the decomposition of iron pentacarbonyl at high temperature. The impact tensile strength, static tensile strength, hardness, absorbability, and frost resistance of the obtained polymer composites were examined. The surface topography of the composites was also examined and their surface roughness, surface water contact angle, and free surface energy were determined. To sum up the problem, the constant magnetic field tended to increase the hardness or impact tensile strength of polymer composites with different admixtures. The change was greater or smaller, depending on the admixture used. The constant magnetic field, with the additional participation of carbonyl iron, caused an increase in the frost resistance of most polymer composites. The surfaces of polymer composites obtained in a constant magnetic field were characterized by greater smoothness and compaction compared to the surface of composites obtained without the presence of a constant magnetic field. The surfaces of the composites obtained without the participation of a constant magnetic field, observed with an optical profilometer, had many depressions and bulges formed by accumulated magnetic particles. In a constant magnetic field, no such bulges were observed, because they were absorbed into the material and arranged along the force lines of the magnetic field. As a result, the polymer composites obtained in a constant magnetic field showed less surface roughness. The highest static tensile strength was shown by a sample made of Epidian 5 resin alone, which is a matrix in the composition, without the action of a magnetic field. Both the Epidian 5 epoxy resin itself and all the composites on its matrix, obtained both in the presence of a magnetic field and without the presence of a magnetic field, showed high wettability of the surface by water, and a high hydrophilicity.

## Figures and Tables

**Table 1:** Epidian 5 epoxy resin-based polymeric composites with or without filling additives and magnetic particles prepared in a constant magnetic field or without a constant magnetic field.

Sample	Filling Additive (20% by wt.)	Magnetic Particle (10% by wt.)	Magnetic Induction B [T]
PR1	Powder graphite 390	-	0.0
PR1′	Powder graphite 390	-	0.5
PR2	Expanded graphite EG 290	-	0.0
PR2′	Expanded graphite EG 290	-	0.5
PR3	Powder graphite 390	Carbonyl iron	0.0
PR3′	Powder graphite 390	Carbonyl iron	0.5
PR4	Expanded graphite EG 290	Carbonyl iron	0.0
PR4′	Expanded graphite EG 290	Carbonyl iron	0.5
PR5	Birch bark with betulin	-	0.0
PR5′	Birch bark with betulin	-	0.5
PR6	Birch bark with betulin	Carbonyl iron	0.0
PR6′	Birch bark with betulin	Carbonyl iron	0.5
PR7	Yellow dextrin	-	0.0
PR7′	Yellow dextrin	-	0.5
PR8	Yellow dextrin	Carbonyl iron	0.0
PR8′	Yellow dextrin	Carbonyl iron	0.5
PR9	-	-	0.0
PR9′	-	-	0.5
PR10	Birch bark with betulin	Carbonyl iron	0.25
PR11	Birch bark with betulin	Carbonyl iron	0.75
PR12	Birch bark with betulin	Carbonyl iron	1.0
PR13	Expanded graphite EG 290	Carbonyl iron	0.25
PR14	Expanded graphite EG 290	Carbonyl iron	0.75
PR15	Expanded graphite EG 290	Carbonyl iron	1.0
PR16	-	Carbonyl iron	0.0
PR17	-	Carbonyl iron	0.5

**Table 2:** Roughness parameters of polymeric composites PR16, PR17, PR6 and PR6′. Composite composition is presented in Table 1.

Parameter	Description	Unit	Sample			
			PR16 (B = 0.0 T)	PR17 (B = 0.5 T)	PR6 (B = 0.0 T)	PR6′ (B = 0.5 T)
Rp	Maximum peak height of the roughness profile	nm	147.1	110.5	149.6	58.3
Rz	Maximum roughness profile height	nm	235.2	198.2	219.2	171.8
Rt	The total height of the roughness profile	nm	278.7	198.2	219.2	171.8
Ra	The deviation of the arithmetic mean of the roughness profile	nm	36.16	22.84	29.06	18.99
Rq	Mean square deviation of the roughness profile	nm	46.39	29.15	37.14	26.09
Rdq	Mean square slope of the roughness profile	grade	1.1070	0.8734	1.0000	0.5413
Rdc	The difference in the height of the roughness profile parts	nm	67.71	47.05	61.31	37.86

## Data Availability

Not applicable.
